# Supplementary material for: Time-Dependent Changes in Hematoma Expansion Rate after Supratentorial Intracerebral Hemorrhage and Its Relationship with Neurological Deterioration and Functional Outcome
Source: Diagnostics (Basel). 2024 Jan 31;14(3):308. doi: 10.3390/diagnostics14030308 (PMC10855868; doi:10.3390/diagnostics14030308)
Supplement: Supplementary file 1 [file diagnostics-14-00308-s001.zip › diagnostics-2829298-supplementary.pdf]

Table S1. Clinical characteristics of patients included in the analysis versus those with supratentorial intracerebral hemorrhage who were excluded due to a baseline CT obtained >24 hours from the onset or follow-up CT >48 hours after the onset (Figure 1 flowchart).

	**Included (n=567)**	**Exlcuded (n=43)**	**P value**
**Male sex**	308 (54.3%)	22 (51.2%)	0.752
**Age** [years]	69.8 ± 14.2	73.2 ± 14.1	0.133
**Ethnicity –** Hispanic	46 (8.1%)	3 (7.0%)	1.00
**Race –**			
White	391 (69%)	33 (76.7%)	
Black	160 (28.2%)	7 (16.3%)	<0.001
Asian	15 (2.6%)	1 (2.3%)	
Other	1 (0.2%)	2 (4.7%)	
**Systolic Blood pressure** [mmHg]	172.1 ± 31.9	160.8 ± 27.5	0.024
**History of hypertension**	478 (84.5%)	35 (81.4%)	0.663
**Baseline blood glucose** [mg/dL]	143.5 ± 61.1	120.8 ± 42.6	0.017
**History of anticoagulation**	115 (20.3%)	8 (18.6%)	1.00
**Onset to baseline CT time gap** [hours]	5.2 ± 5.9	16.6 ± 11.5	<0.001
**Baseline hematoma volume** [mL]	20.2 ± 21.6	18.8 ± 19.9	0.700
**Baseline to follow-up CT time gap** [hours]	18.3 ± 16.2	19.7 ± 15.5	0.608
**Follow-up hematoma volume** [mL]	25.3 ± 27.3	19.1 ± 19.3	0.146
**Admission Glasgow Coma Scale score**	14 (10 – 15)	15 (14 – 15)	0.068
**Admission NIH Stroke Scale score**	10 (4 – 18)	2 (1 – 7)	<0.001
**Deep (vs lobar) hemorrhage**	332 (58.6%)	14 (32.6%)	0.001
**Intraventicular hemorrhage**	290 (51.1%)	18 (41.9%)	0.270
**External ventricular drainage**	66 (11.6%)	1 (2.3%)	0.073
**Surgical evacuation**	29 (5.1%)	2 (4.7%)	1.00
**Hematoma Expansion**	177 (31.2%)	8 (18.6%)	0.088
**Neurological Deterioration ***	212 (37.4%)	13 (30.2%)	0.414
**3-month outcome (modified Rankin Score)**	4 (2 – 6)	3 (1 – 4)	0.002
**Mortality**	159 (28.0%)	6 (14.0%)	0.050

* Neurological deterioration defined by ≥4-point increase in NIHSS or ≥2-point drop in GCS in the first week of admission.
